# Post-transplant de-novo renal phospholipidosis in a kidney transplant recipient: Fabry disease or something else? 

**DOI:** 10.5414/CNCS110131

**Published:** 2020-05-29

**Authors:** Muhammad Saad Naseer, Raj Chand, Stefano Coppola, Adrian Abreo, Mukesh Sharma, Neeraj Singh

**Affiliations:** 1 John C. McDonald Regional Transplant Center – Willis Knighton Health System,; 2 Department of Internal Medicine, Division of Nephrology, Louisiana State University Health Sciences Center (LSUHSC), Shreveport, LA, and; 3 Department of General and Interventional Nephrology, Sierra Nevada Nephrology Consultants, Reno, NV, USA

**Keywords:** renal phospholipidosis, lamellar inclusions, cationic amphiphilic drugs, Fabry disease, myelin bodies

## Abstract

Renal phospholipidosis is a rare cause of proteinuria and kidney dysfunction. We describe a kidney transplant recipient who presented with slowly rising serum creatinine, nephrotic range proteinuria, and lower extremity edema 10 years post transplant. He was diagnosed with renal phospholipidosis on the transplant kidney biopsy. Patient did not have prior history or current symptoms or signs of Fabry disease. Serum α-galactosidase level was normal. The etiology was suspected to be due to chronic use of sertraline, a previously reported cause of drug-induced renal phospholipidosis. Sertraline was discontinued, and proteinuria declined with stabilization of kidney function at 6-months follow-up.

## Introduction 

Drug-induced renal phospholipidosis (DIP) post kidney transplant has not been previously reported. We report a case of sertraline-induced renal phospholipidosis and discuss several other etiologies that may result in renal phospholipidosis mimicking Fabry disease. It is important to recognize DIP, as discontinuation of suspected drug may reverse the pathological process and improve outcomes. 

## Case description 

**Figure 1:**
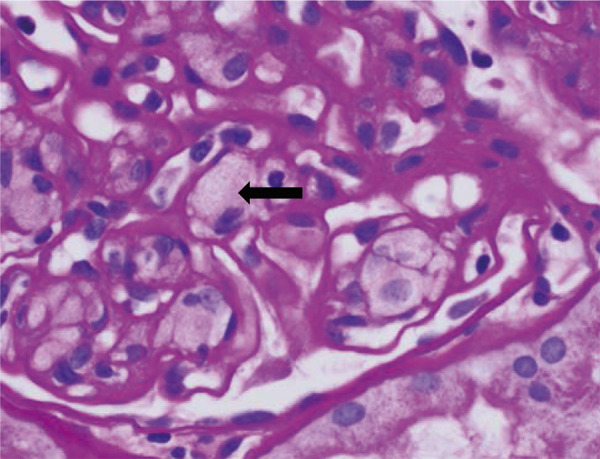
The H & E stain of transplant kidney biopsy done 10 years post transplantation shows enlarged glomerular capillary endothelial cells with foamy cytoplasm (black arrow).

**Figure 2:**
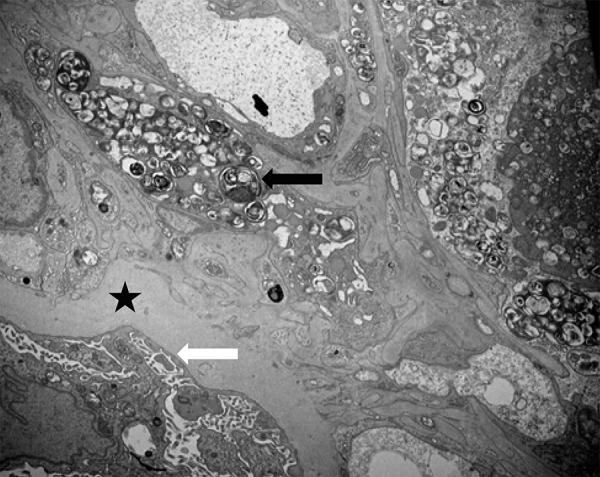
Electron microscopy of transplant kidney biopsy done 10 years post transplantation shows an endothelial and mesangial cell with numerous lamellar and dense cytoplasmic inclusions (myelin bodies) (black arrow). Glomerular capillary basement membrane is thickened (marked by star), and effacement of podocyte foot processes is present (white arrow).

A 63-year-old Asian male with prior history of kidney transplant 10 years ago due to end-stage renal disease secondary to hypertension presented with lower extremity edema for 2 weeks. A month post transplant, he had an episode of biopsy-proven rejection but no complications otherwise. His maintenance immunosuppression consisted of mycophenolate mofetil 750 mg oral twice daily, tacrolimus 3 mg oral twice daily, and prednisone 2.5 mg oral once daily. In addition, the patient had been on sertraline 200 mg oral once daily, nifedipine 10 mg oral once daily, and vitamin D3 1,000 U oral once daily. On examination, his vitals were stable, and examination was unremarkable except for 2+ pedal edema. Laboratory data showed a slowly rising serum creatinine over the past 6 months with current value of 2.3 mg/dL (baseline 1.5 – 1.8 mg/dL), a spot urine protein-to-creatinine ratio of 7.6 g/g of creatinine, and tacrolimus level of 4.7 ng/mL. BK virus PCR and donor-specific anti-HLA antibodies were negative. The patient had a spot urine protein-to-creatinine ratio of 0.9 g/g of creatinine 6 months prior. The transplant kidney biopsy showed focal mild interstitial fibrosis with tubular atrophy, glomeruli with lobulation of tufts, large endothelial cells with foamy cytoplasm (Figure 1), glomerular capillary endothelial cells, and mesangial cells containing lamellar and dense cytoplasmic inclusions or myelin bodies (Figure 2). No rejection or viral cytopathic effects, immune complex deposits, or fibrils were identified. The stains for polyomavirus and for C4d were negative. In addition to chronic transplant glomerulopathy, the diagnosis of glomerular phospholipidosis was entertained. The serum α-galactosidase A level was normal, 0.136 U/L (reference range: 0.074 – 0.457). Sertraline was discontinued and patient was switched to bupropion. The proteinuria declined to 2.3 g/g of creatinine with stabilization of serum creatinine at 6-months follow-up visit. 

## Discussion 

Lysosomes are an important site for the catabolism of phospholipids by different phospholipase enzymes. The inhibition of the activity of phospholipases leads to intracellular accumulation of phospholipids which presents as foamy cytoplasm, evident in Figure 1. On electron microscopy, the development of concentric lamellar bodies, also called myelin or zebra bodies, can be appreciated in detail, which is the ultrastructural hallmark of renal phospholipidosis, as shown in Figure 2. 

Fabry disease is a well-known cause of renal phospholipidosis and is caused by a genetic deficiency of lysosomal enzyme α-galactosidase A, which results in progressive accumulation of glycosphingolipids within different body cells. Fabry disease is associated with renal and extra-renal manifestations of angiokeratomas, hypohidrosis, hearing loss, corneal opacity, neurological and cardiac involvement. Renal lamellar inclusions in Fabry disease are ultrastructurally similar to those seen in acquired causes of phospholipidosis. The diagnosis of Fabry disease is suggested by typical clinical signs and symptoms and confirmed by low enzyme activity in peripheral blood or in leukocytes, or by genetic mutation analysis. Our patient had no clinical signs and symptoms suggestive of Fabry disease and his serum α-galactosidase A level was normal. Hence, genetic analysis was not ordered. In Fabry patients, recurrence in allograft post kidney transplant from non-Fabry donors is rare and graft survival is not reduced as compared with patients with other causes of end-stage renal disease, but the risk of death post transplant is higher [1]. Niemann-Pick disease, another lipid storage disorder results either due to the deficiency of lysosomal enzyme, acid sphingomyelinase or due to impaired movement of lipids within cells. It primarily affects children, and kidneys are rarely involved [2]. 

Besides Fabry and Niemann-Pick disease, renal phospholipidosis has also been reported in silicosis [3] and due to several drugs [4] including amiodarone, chloroquine, aminoglycosides, chlorpromazine, fluoxetine, sertraline, and azithromycin. DIP is a form of acquired lysosomal storage disease. The cationic amphiphilic drugs (CADs) enter the lysosomes and inhibit the activity of phospholipase, leading to intracellular accumulation of phospholipids. Phospholipidosis induction by drugs is dose dependent, and pathological lesions may reverse after drug withdrawal [5]. One of the views is that DIP is an adaptive response to exposure to CADs and is not a toxic response per se. By this theory, drugs are trapped within lysosomes and sequestered from other cellular sites where they may be toxic [4]. Lungs, liver, and other organs may be affected besides kidneys [4]. DIP in the kidneys is rarely reported due to several reasons. The intracellular accumulation of phospholipids is a slow process, and the use of offending drugs may have to continue for several months to years before the lesions become apparent on biopsy. The DIP lesions are focal, and the pathological changes may escape detection due to sampling error on kidney biopsy. Electron microscopy is necessary to detect the hallmark ultrastructural DIP lesions but is not ordered routinely. Furthermore, patients with CKD and proteinuria may have concomitant hypertension and/or diabetes mellitus, and kidney biopsy is not routinely performed in these conditions. 

In summary, DIP is a rare cause of renal dysfunction and nephrotic range proteinuria post kidney transplantation and should be considered in the differential diagnosis in patients presenting with dense cytoplasmic inclusion (myelin) bodies on kidney biopsy after excluding recurrent Fabry disease. 

## Funding 

None. 

## Conflict of interest 

None.
